# Emerging frontiers in aromaticity

**DOI:** 10.1039/d3sc90163g

**Published:** 2023-09-07

**Authors:** Miquel Solà, Israel Fernández, Gabriel Merino

**Affiliations:** a Institut de Química Computacional i Catàlisi and Department de Química, Universitat de Girona C/ M. Aurèlia Capmany, 69 Girona 17003 Catalonia Spain miquel.sola@udg.edu; b Departamento de Química Orgánica and Centro de Innovación en Química Avanzada (ORFEO-CINQA), Facultad de Ciencias Químicas, Universidad Complutense de Madrid 28040 Madrid Spain israel@quim.ucm.es; c Departamento de Física Aplicada, Centro de Investigación y de Estudios Avanzados, Unidad Mérida km 6 Antigua carretera a Progreso, Apdo. Postal 73, Cordemex Mérida 97310 Yuc. Mexico gmerino@cinvestav.mx

## Abstract

In this themed collection, we embark on a captivating journey into the realm of aromaticity, a fundamental concept that has attracted chemists for nearly two centuries. This virtual collection offers a comprehensive overview of the recent advances in the field, encompassing thirty manuscripts published in Chemical Science from 2021 to the present. Aromaticity, a concept with a rich history has undergone substantial evolution. Its significance transcends the boundaries of organic chemistry, expanding its influence into the domains of inorganic chemistry, organometallic chemistry, and materials science. This collection shows the dynamic nature of contemporary research within this fascinating field.

## Abstract 2

This themed collection reflects on the relevance of aromaticity in modern chemistry by highlighting the most recent discoveries and views, offering a selection of articles on a wide variety of relevant subjects.

## Graphical abstract

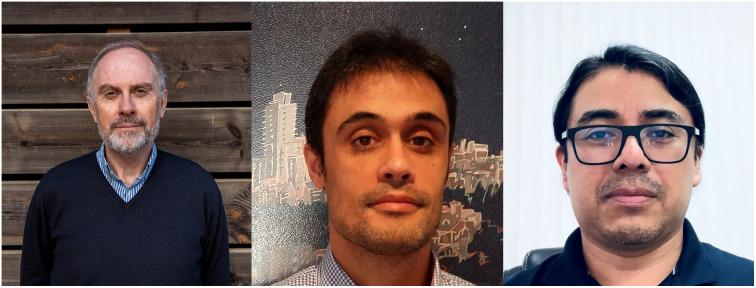

In the 2023 issue 21 of *Chemical Science*, we, along with ten other authors working on different aspects related to aromaticity, published a Perspective entitled “Aromaticity: Quo Vadis” (https://doi.org/10.1039/D2SC04998H). In this Perspective, we explored several controversial aspects of the aromaticity concept, generating considerable interest within the chemistry community and reaffirming that aromaticity remains one of the most intriguing and captivating concepts in the field. Consequently, we were tasked with curating a virtual collection highlighting thirty papers published in *Chemical Science* since 2021. This task proved challenging, given the substantial number of papers (262 according to Web of Science, accessed on the 21st July 2023) containing the term “aromatic*” in their title, abstract, or keywords. To ensure a comprehensive representation of the current topics in aromaticity, we applied the criterion that corresponding authors could only appear once in the collection.

Among the notable inclusions in this collection is the Focus article by Ottosson, which raises essential crucial questions not explored in our Perspective (https://doi.org/10.1039/D3SC90075D). Ottosson ponders who uses the aromaticity concept and benefits from its ambiguous definition, and conversely, who might seek more precision.

The term “aromatic” was first employed by Hofmann one hundred and fifty years ago to describe a group of compounds with properties like benzene.^1^ Since then, this concept has undergone a significant evolution. Recent years have witnessed numerous papers dedicated to developing new methodologies for analyzing aromaticity and investigating other manifestations of aromaticity such as metalla-aromaticity, macrocyclic aromaticity, 3D-aromaticity, Möbius aromaticity, and aromaticity of polycyclic conjugated hydrocarbons (PCHs) and nanographenes, including species with chiral properties and polyradical character. Our selection encompasses work covering all these subjects.

This collection presents new methodological advancements, including contour maps of isotropic magnetic shielding (IMS) (https://doi.org/10.1039/D1SC03368A) which proved especially valuable for analyzing aromaticity in contorted polycyclic aromatic hydrocarbons (PAHs); and a method employing the Biot–Savart law to deconvolute the contributions of different ring currents to the experimental NMR spectra of PAHs (https://doi.org/10.1039/D2SC05923A).

Within the realm of metalla-aromaticity, this collection includes seven papers. One of them examines the chemical bonding in the smallest 4f-metalla-aromatic species (cyclo-PrB_2_^−^), displaying both σ- and π-aromaticity (https://doi.org/10.1039/D2SC02852B). Additionally, two papers report the synthesis of spiro-metalla-aromatic systems, (https://doi.org/10.1039/D0SC04469E, https://doi.org/10.1039/D2SC05378K), while another explores the interaction of the cluster Ag_4_^2+^ with the human copper chaperone Atox1 (https://doi.org/10.1039/D1SC07122J). Furthermore, two papers investigate the reactivity of metalla-aromatic compounds, (https://doi.org/10.1039/D1SC01571K, https://doi.org/10.1039/D2SC05455H), and a contribution discusses the Möbius metalla-aromatic character of an interlocked Mn_2_B_10_H_10_ wheel (https://doi.org/10.1039/D2SC02244C).

The collection also gathers four papers focused on macrocyclic aromaticity. For instance, trioxopyrrolocorphins exhibit unexpected macrocyclic aromatic properties (https://doi.org/10.1039/D1SC03403K). The fusion of benzoimidazo-isoindole on the porphyrin ring coordinated to C_60_, results in a long-lived charge-separated state (https://doi.org/10.1039/D2SC03238D) while four-electron addition to [8]cycloparaphenylene ([8]CPP) leads to significant structural deformation (https://doi.org/10.1039/D1SC00713K).

The intriguing phenomenon of chiral-induced spin selectivity (CISS) has opened new possibilities for using chiral compounds in spintronics applications and gaining a deeper understanding of spin-selective biological processes. Consequently, the study of chiral PCHs and PAHs is currently a hot topic. Within our selection, readers will find papers on chiral dihetero[8]helicenes (https://doi.org/10.1039/D1SC00044F) BN-[*n*]helicenes (*n* = 5, 6) (https://doi.org/10.1039/D1SC06513K) and buckybowls connected to helicene units (https://doi.org/10.1039/D3SC00658A). Of particular interest, Esser *et al.* show the generation of enantiopure nanohoops through racemic resolution of diketo[*n*]cycloparaphenylenes (https://doi.org/10.1039/D1SC02718B). Also, one paper explores placing Mg-porphyrin molecules in a chiral optical cavity to observe enantiomer-selective photochemical processes (https://doi.org/10.1039/D1SC04341B).

Nanographenes and PCHs are also prominently featured in this collection, being among the most studied species. Their ability to attain diradical character makes them attractive targets in the quest for systems with low-lying triplet states (https://doi.org/10.1039/D3SC01295F, https://doi.org/10.1039/D0SC04699J). Furthermore, curved nanographenes are anticipated to possess unique physicochemical properties, achieved by introducing nonhexagonal rings (*e.g.* pentagonal, heptagonal, and octagonal rings) to create curved (aza-)nanographenes (https://doi.org/10.1039/D2SC05858H, https://doi.org/10.1039/D2SC04722E, https://doi.org/10.1039/D1SC05586K). Additionally, doped nanographenes offer opportunities to generate species with interesting properties, as exemplified by a novel family of boraolympicenes (https://doi.org/10.1039/D3SC00342F). Lastly, one paper in the collection examines the substituent effects on the ring currents of π-extended hexapyrrolohexaazacoronene (https://doi.org/10.1039/D2SC07037E) while another reports the violation of the Kasha photoemission rule in a PCH (https://doi.org/10.1039/D3SC00405H).

The topic of 3D-aromaticity is also addressed in the context of carboranes fused to 2D-aromatic rings, revealing that the aromatic character of the 2D rings diminishes upon fusion with the carborane (https://doi.org/10.1039/D2SC03511A). Finally, two papers delve into the interactions of aromatic molecules with anions in the Protein Data Bank (https://doi.org/10.1039/D2SC00763K) and the substituent effects on the aromatic interactions in water (https://doi.org/10.1039/D3SC01027A).

As guest editors, we consider this compilation of articles offers a comprehensive view of the current state-of-the-art in aromaticity research. We hope that the papers collected in this collection will serve as a source of inspiration not only for researchers in the field but also to most readers of *Chemical Science*.

## Supplementary Material
